# Sequence analysis and confirmation of the type IV pili-associated proteins PilY1, PilW and PilV in *Acidithiobacillus thiooxidans*

**DOI:** 10.1371/journal.pone.0199854

**Published:** 2019-01-07

**Authors:** Elvia Alfaro-Saldaña, Araceli Hernández-Sánchez, O. Araceli Patrón-Soberano, Marizel Astello-García, J. Alfredo Méndez-Cabañas, J. Viridiana García-Meza

**Affiliations:** 1 Geomicrobiología, Metalurgia, Universidad Autónoma de San Luis Potosí, San Luis Potosí, México; 2 Biofísica Molecular, Instituto de Física, Universidad Autónoma de San Luis Potosí, San Luis Potosí, México; 3 División de Biología Molecular, Instituto Potosino de Investigación Científica y Tecnológica, San Luis Potosí, México; Nazarbayev University, KAZAKHSTAN

## Abstract

*Acidithiobacillus thiooxidans* is an acidophilic chemolithoautotrophic bacterium widely used in the mining industry due to its metabolic sulfur-oxidizing capability. The biooxidation of sulfide minerals is enhanced through the attachment of *At*. *thiooxidans* cells to the mineral surface. The Type IV pili (TfP) of *At*. *thiooxidans* may play an important role in the bacteria attachment since TfP play a key adhesive role in the attachment and colonization of different surfaces. In this work, we report for the first time the mRNA sequence of three TfP proteins from *At*. *thiooxidans*, the adhesin protein PilY1 and the TfP pilins PilW and PilV. The nucleotide sequences of these TfP proteins show changes in some nucleotide positions with respect to the corresponding annotated sequences. The bioinformatic analyses and 3D-modeling of protein structures sustain their classification as TfP proteins, as structural homologs of the corresponding proteins of *Ps*. *aeruginosa*, results that sustain the role of PilY1, PilW and PilV in pili assembly. Also, that PilY1 comprises the conserved *Neisseria*-PilC (superfamily) domain of the tip-associated adhesin, while PilW of the superfamily of putative TfP assembly proteins and PilV belongs to the superfamily of TfP assembly protein. In addition, the analyses suggested the presence of specific functional domains involved in adhesion, energy transduction and signaling functions. The phylogenetic analysis indicated that the PilY1 of *Acidithiobacillus* genus forms a cohesive group linked with iron- and/or sulfur-oxidizing microorganisms from acid mine drainage or mine tailings.

## Introduction

*Acidithiobacillus thiooxidans* is an acidophilic chemolithoautotroph that uses reduced sulfurs as a source of electrons and reducing power, including elemental sulfur (S^0^), polysulfides (S_*n*_^2-^) and sulfide minerals, such as pyrite (FeS_2_), chalcopyrite (CuFeS_2_) or sphalerite (ZnS).

Bacterial attachment to mineral surfaces influences the rate of dissolution of the mineral because of surficial phenomena including decreases in mixed potentials as well as changes in kinetics and mass-transport processes [1]. Hence, bacterial attachment is due to self-organization by a bioelectrochemical evolution on the interface. Interfacial studies on charge and mass transfer demonstrate that S^0^ biooxidation by *At*. *thiooxidans* begins in the early stages of the interaction (1 to 24 h) when the biofilm is still not constituted. Moreover, S^0^ biooxidation is primarily controlled by surficial characteristics that pivoted the bacterial attachment to the hydrophobic S^0^; such attachment is an energy-dependent process in which *At*. *thiooxidans* essentially activates or modifies the reactive properties of S^0^ [2, 3]. Furthermore, the hydrophobic character of the interface “determines the free energy of the adhesion process” [4]. The Type IV pili (TfP) of *At*. *thiooxidans* may play an important role in the bacterial attachment and bioelectrochemical evolution on the bacteria-mineral interface, *e*.*g*., surpassing hydrophilic interactions.

Valdés *et al*. [5] and Li *et al*. [6] suggested that the efficiency of *At*. *ferrooxidans* to attach and colonize mineral surfaces (*e*.*g*., FeS_2_ or CuFeS_2_) and solid reduced sulfur depends on TfP as well as on its multiple copies of genes for pili biosynthesis. Other *Acidithiobacillus* species such as *At*. *caldus* and *At*. *thiooxidans* contain several genes coding for TfP assembly proteins [7–9] that are related to biofilm formation, acting as c-di-GMP effector proteins [10].

TfP are semiflexible polymeric filaments of pilins anchored to the cellular membrane. They are 5–7 nm in diameter and range from 4–5 μm up to several micrometers in length [11,12]. TfP have been grouped based on the aminoacidic homology of the pilin subunits, which are relatively conserved in prokaryotes. Pilins share an N-terminal cleavage/methylation (N-methylphenylalanine) domain (NTD) of approximately 25 amino acids (aa) followed by a stretch of hydrophobic residues forming an extended α-helix and a disulfide bond at the C-terminal domain (CTD) [11, 13]. Pilins interact via their conserved NTD α-helix, which forms a hydrophobic core that provides extreme mechanical strength [13]. Furthermore, it has been reported that this conserved NTD in prepilins is also present in the type II secretion system (T2SS), also known as pseudopilins [14–17].

Pelicic [18] suggested that the minimal machinery needed for TfP assembly includes pilins and other TfP proteins: (i) a Major (structural) pilin bearing a NTD (pilin-like motif), (ii) a specific peptidase that processes the precursors of pilins or prepilins (*e*.*g*., PilD of *Neisseria* spp.), (iii) a traffic ATPase that powers TfP assembly (PilT), (iv) an internal inner membrane protein (PilG), (v) an integral outer membrane necessary for the emergence of TfP on the cell surface, a secretin (PilQ), and (vi) proteins also found in T2SS, the pilin-like proteins.

Interesting, “TfPs are universal in prokaryotes and have shown extreme functional versatility”. Among other functions (motility, cell signaling, pathogenic functions, protein secretion, DNA uptake, electrical conductance, and so on), TfP are “sticky organelles” that play a key role as adhesive to stick bacteria to one another and to attach to and to colonize a wide variety of surfaces, leading to the formation of colonies and even biofilms with cells embedded within the extracellular polymeric substances (EPS) matrix, including “2-D” (thin liquid) and 3-D biofilms [12, 17].

The adhesive ability of TfP is due to the presence of a non-pilin protein, an adhesin (integrin) located on the distal tip of the TfP [17, 19–21]. This adhesin, designated PilC or PilY1, is expressed in multiple species. For instance, PilY1 of *Pseudomonas aeruginosa* is the orthologue of the meningococcal PilC of *Neisseria gonorrhoeae*, and although PilY1 and PilC share only a partial sequence homology, they have a structural similarity (*e*.*g*., 46% similarity and 24% identity) [22,23]. PilC is a phase-variable protein that belongs to a conserved protein domain family of several PilC protein sequences [24]. Further, PilC/PilY1 is dispensable for TfP assembly at early stages of TfP biogenesis, for instance, in the absence of pilus retraction [17, 23–25]. Moreover, the tip-associated adhesin PilY1 of *At*. *thiooxidans* Licanantay (WP_031573362] exhibits 55% of identity with the type IV pilin biogenesis protein of *At*. *thiooxidans* ATCC 19377 (WP_010638975.1) and 86% of identity with that of *At*. *albertensis* (WP_075322776.1).

Other proteins involved in adhesion are the minor, but highly conserved pilin proteins, PilW and PilV of *Neisseria* spp. [17, 25, 26]. The outer-membrane lipoprotein PilW participates in the pilus biogenesis for the stabilization of the pilus but not for their assembly and allows bacterial adherence, as well [27]. Another possible function for PilW is in the active transfer of electrons, as has been proposed for *At*. *ferrooxidans* PilW [28] and other species with TfP [29].

Because most genes of *At*. *thiooxidans* are annotated genomic sequences reported to GenBank, but their actual expression has not been yet confirmed. In this work, the expression of type IV pili of *At*. *thiooxidans* was determined, and the mRNA for the putative proteins PilY1, PilW and PilV were identified and sequenced (S1 Table). Moreover, bioinformatic analyses and 3-D modeling of each of these TfP proteins show that PilY1 is an adhesin whereas PilW and PilV are pilus assembly proteins (structural pilins) of the TfP.

## Materials and methods

### Acidithiobacillus thiooxidans culture and maintenance

The type strain of *At*. *thiooxidans* ATCC 19377 [30] was cultured in ATCC 125 medium (in g/L: 0.4 (NH_4_)_2_SO_4_, 0.5 MgSO_4_·7H_2_O, 0.25 CaCl_2_, 3 KH_2_PO_4_, 0.005 FeSO_4_·7H_2_O, and 10 S^0^, pH 2.0 adjusted with concentrated H_2_SO_4_). Cultures were aerobically incubated at 29 ± 1°C under orbital agitation (110 to 120 rpm) for 5, 15 and 21 days.

### Multiplex RT-PCR and sequencing of pilY1, pilW, and pilV

All primers for RT-PCR and alignment analysis were designed using the *At*. *thiooxidans* ATCC 19377 genome (GenBank accession number AFOH01000001) originally described by Valdés et al. [7]. Accordingly, derived annotated protein sequences for PilY1, PilV and PilW were also taken from GenBank under the accession numbers WP_010638975.1, WP_010638981.1 and WP_010638979.1, respectively. Likewise, derived-specific gene sequences for *pilY1* (ATHIO_RS0106065), *pilW* (ATHIO_RS0106075) and *pilV* (ATHIO_RS0106080) were also taken from the GenBank database. Primers were designed using the free internet-based software Primer3 [31] and confirmed using Vector NTI (InforMax-Invitrogen, USA). As a positive control for each RT-PCR reaction, a specific pair of primers to amplify the expression of the 16S rRNA (AJ459803) was designed.

Total RNA was extracted from 5, 15 and 21 days cultures. Briefly, cells were collected by centrifugation at 0.08 x g for 1 minute to separate the cells from S^0^. Cells were then concentrated and washed by centrifugation at 21.1 x g for 1 min using saline phosphate buffer (PBS; in g/L: 8 NaCl, 1.44 Na_2_HPO_4_, 0.24 KH_2_PO_4_ (J.T. Baker, USA), and 0.2 KCl (Sigma-Aldrich, USA); pH 7.4).

RNA extraction was performed using TRIzol reagent (Invitrogen, USA) according the manufacturer's protocol. After quantification using a NanoDrop 2000c (Thermo Scientific, USA), cDNA synthesis was performed according to the manufacturer’s recommendation, using 200U M-MLV (Invitrogen, USA) in a 20 μL total volume reaction. The amplification products were purified using the QIAquick kit (Qiagen, USA) following the recommended procedure, quantified and sequenced. The products were sequenced by the Sanger method at LANBAMA (National Laboratory of Biotechnology, San Luis Potosí, Mexico) and LANGEBIO (National Laboratory of Genomic for the Biodiversity, Guanajuato, México) facilities. Each sequence was confirmed at least five times by analyzing amplification products obtained from different culture replicates. The generated sequences were compared with the annotated sequences of each corresponding gene using the Multalin software [32].

### In silico (bioinformatic) analyses of proteins

The confirmed sequences of PilY1, PilW and PilV were analyzed to search for domains, families and functional sites with bioinformatic tools, including Simple Modular Architecture Search, SMART [33], PROSITE [34], and EMBL-EBI [35] and the CDD/SPARCLE domain analysis function [36]. The protein structure homology-modeling was achieved by using the server SWISS-MODEL [37]. The 3-D model of each protein was generated in I-TASSER [38, 39]. The analyses of possible ligands were performed in the Ligand-Protein Binding Database, BioLip [40].

### Phylogenetic relationships

Phylogenetic relationships were analyzed for the confirmed nucleotide and protein sequences of PilY1, PilW and PilV from *At*. *thiooxidans* ATCC19377 against those of the NCBI database using BLASTN 2.8.0+ [41, 42], and UniProt [43] bioinformatic resources. For PilY1, we also included the sequence of PilC of *Neisseria meningitidis* (WP_101069668.1) and of PilY1 of *Ps*. *aeruginosa* PAO1 (AAA93502.1) because according to the bioinformatic analysis, the confirmed sequence of PilY1 of *At*. *thiooxidans* comprise the Neisseria-PilC superfamily domain [34] and shares sequence and structural homologies with the CTD of PilY1 of *Ps*. *aeruginosa* (3hx6.1) [23].

The sequences obtained from NCBI were aligned with those obtained in this work using MEGA7 software [44] by the Neighbor-Joining method. Thus, the evolutionary history was inferred by using the Maximum Likelihood method (ML) based on the Jones-Taylor-Thornton (JTT) model, in which the JTT-distances corrects for multiple substitutions based on the model of amino acid substitution (substitution-rate JTT-matrix), allowing to perform a recounting of the number of observed changes [45]; for the bootstrap consensus tree, 1000 replicates were performed [46]. The percentage of trees in which the associated taxa clustered together is shown next to the branches. Initial trees for the heuristic search were obtained automatically by applying Neighbor-Join and BioNJ algorithms to a matrix of pairwise distances estimated using a JTT model; the tree with the highest log likelihood is shown. All positions with less than 95% site coverage were eliminated.

### Transmission electron microscopy (TEM) analyses

After 5 days of incubation, *At*. *thiooxidans* cells were negative stained with 1% phosphotungstic acid (PTA) or 2% uranyl acetate. One aliquot was directly used, and the other was fixed previously to reduce the insoluble S^0^ present in the sample and stabilize pili. Both samples were washed three times at 1500 rpm for 5 min and stained with PTA. The pili were observed using TEM (JEOL 200CX, Japan) at 100 kV.

## Results and discussion

### TEM analyses

**Fig 1:**
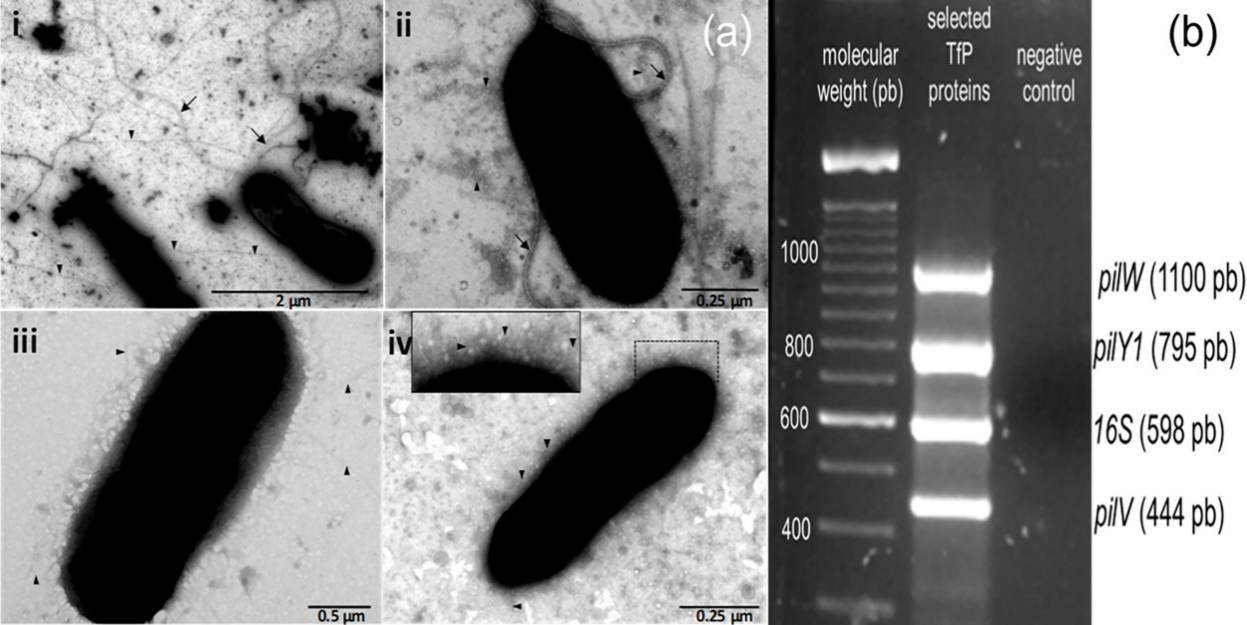
Identification of pili in *At*. *thiooxidans*. Representative micrographs of pili (cell negative staining) are shown in (**a**); amplification of mRNA coding for the pilus protein *pilY1*, *pilW*, and *pilV* are shown in (**b**). Cells were negatively stained with 2% uranyl acetate (i) or 1% phosphotungstic acid (ii-iv). (iii) represents cells after wash while (iv) represents cells after both wash and fixation prior to PTA staining, respectively. Pili are pinpointed by triangles whereas flagella are by arrows. The expression of *pilY1*, *pilW*, and *pilV* was evaluated by multiplex RT-PCR using total RNA obtained from cells after 5 days of culture. The 16S rRNA was used as internal positive control. Water instead of RNA was used as negative control. Identity of each PCR product was confirmed by sequencing.

The type IV pili (TfP) of *At*. *thiooxidans* may play an important role in the bacterial attachment to mineral surfaces, leading to an increase in the rate of dissolution of sulfide minerals. Considering that the genome of the type strain of *At*. *thiooxidans* ATCC 19377 [30] contains genes coding for the assembly of TfP [7–9], we first evaluated the presence of pili on its bacterial surface. Cell of 5 days in culture were negative stained with 2% uranyl acetate or 1% phosphotungstic acid (PTA). As can be seen in Fig 1A, negative staining with 2% uranyl made both the flagellum and pili visible (i), however, S^0^ was visible too. We next performed a negative staining with 1% PTA, under these conditions, the pili and flagellum were covered by extracellular polymeric substances (EPS) (ii), to prevent that, prior to the staining with 1% PTA cells were washed, although both S^0^ and EPS were eliminated, flagella and most of pili were lost as well. In order to avoid this, bacterial cells were fixed before washing, which allowed us to detect well-preserved pili in an arranged network on the cell surface (iv, insert). We thus conclude that after microscopic analyses, pili were identified in all tested conditions.

After the presence of pili in *At*. *thiooxidans* was confirmed, we proceeded to evaluate the expression of the mRNA for the proteins forming the pili at different culture times (5, 15 and 21 days; data not show). The multiplex RT-PCR to detect the expression of the adhesin *pilY1* as well as the major structural pilin *pilW* and of the pilus assembly protein *pilV* were performed in cells cultured for 5 days. As can be seen in Fig 1B, the mRNAs for all three pilins could be detected; as internal control for the RT-PCR reactions, the 16S rRNA was selected.

### Sequencing analysis

**Fig 2:**
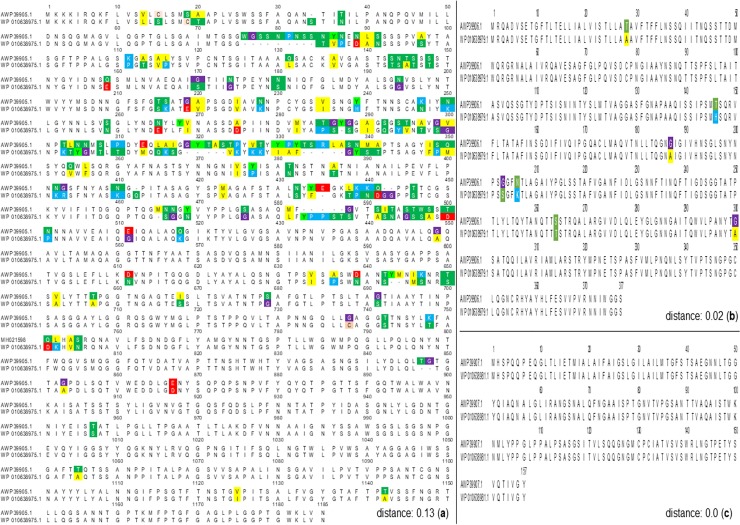
Comparation between the confirmed and annotated sequences of PilY, PilW and PilV at the aminoacidic level. Using the multiple sequence alignment accuracy and high throughput (MUSCLE), the confirmed aminoacidic sequences of PilY1 (AWP39905.1) (**a**); PilW (AWP39906.1) (**b**) and PilV (AWP39907.1) were compared against their respective annotated sequence reported in the GenBank: WP_010638975.1 (**a**), WP_010638979.1 (**b**) and WP_010638981.1. Conserved sites at 100% level are shown in colors. Computed pairwise distance (Poisson model) are also shown. All analyses were performed using MEGA7 software [44].

Once the expression of *pilY1*, *pilW* and *pilV* was confirmed, and considering that no previous report of the coding sequence (CDS) for these genes is available so far, we decided to obtain the whole sequence for each mRNA. Using the annotated sequence of the genome for *At*. *thiooxidans* ATCC 19377 (GenBank AFOH01000001) and of its homologue *At*. *ferrooxidans* ATCC 53993 (GenBank NC_011206.1), we identified conserved regions of *pilY1*. Eight pairs of primers (*pilY-1* to *pilY-8*, S2 Table), whose PCR products were designed to overlap, were used to amplify, purify and sequence each conserved region. The obtained overlapping sequences were aligned against the annotated sequence of the *pilY1* gene (ATHIO_RS0106065). Because several changes between our sequencing analysis and the annotated sequenced were found (highlighted in Fig 2), three new pairs of primers were designed (*pilY-9* to *pilY-11*, S2 Table) to assess if these highlighted changes were *bonafide*. These pairs of primers cover the regions with all insertions and deletions we found. Once the new sequences were obtained, they were individually aligned to their corresponding fragment in the newly assembled sequence. Thus, all changes were confirmed.

A similar strategy was performed for the sequencing of *pilW* and *pilV*; 5 and 4 pairs of primers were designed for *pilW* and *pilV*, respectively (S2 Table). Obtained sequences were aligned and compared to the corresponding annotated sequence (ATHIO_RS0106075 for *pilW* and ATHIO_RS0106080 for *pilV*). All sequences were then reported to the GenBank under the accession numbers MH021598.1 for *pilY1*, MH021599.1 for *pilW* and MH021600.1 for *pilV*.

Our sequencing analysis shows that all three nucleotide sequences show changes with respect to the corresponding annotated genomic sequences (S1 Fig). The changes are more significant for *pilY1*, ought to several changes in bases as well as 15 insertions/deletions, the overall homology is of 86.6%. However, within the first 1560 bp the difference between the annotated and our confirmed sequences is of approximately 25%, whereas in the rest of the sequence the differences account for only 4.1% (S1 Fig). In line with this, when compared at the aminoacidic level, the annotated (WP_010638975.1) and confirmed protein (AWP39905.1) sequences showed a pairwise distance of 0.13 (Fig 2A). In detail, our confirmed PilY1 shares 85% of identity with the annotated sequence reported in GenBank (1007 aa in identical position). Moreover, our PilY1 (AWP39905.1) shares a 99% homology with the sequence of the type IV pilin biogenesis protein of *At*. *thiooxidans* ZBY (WP_065968128.1) and of 77% with the same annotated protein of *At*. *ferrooxidans* BY0502 (WP_064218310.1).

On the other hand, our sequence analysis of *pilW* (MH021599.1) shows that 20 bases are changed with respect to the annotated (ATHIO_RS0106075) sequence (S1 Fig). This is reflected at the protein level as AWP39906.1 shows a 98% of identity with its respective annotated protein sequence (WP_010638979.1) (Fig 2B). Furthermore, AWP39906.1 the PilW confirmed protein sequence of *At*. *thiooxidans* shows 83% homology with its homologous in *At*. *ferrooxidans* ATCC 23270 (WP_064218312.1).

Finally, our *pilV* sequence (MH021600.1) exhibited few changes with respect to its annotated nucleotide sequence (ATHIO_RS0106080). This results in an identical protein when compared to the annotated sequence (WP_010638981.1 (Fig 2C). When compared to its homologous protein in *At*. *ferrooxidans* ATCC 23270 (ACK80286.1), both protein sequences share a 92% of identity.

### Bioinformatic and phylogenetic analyses

#### PilY1

**Fig 3:**
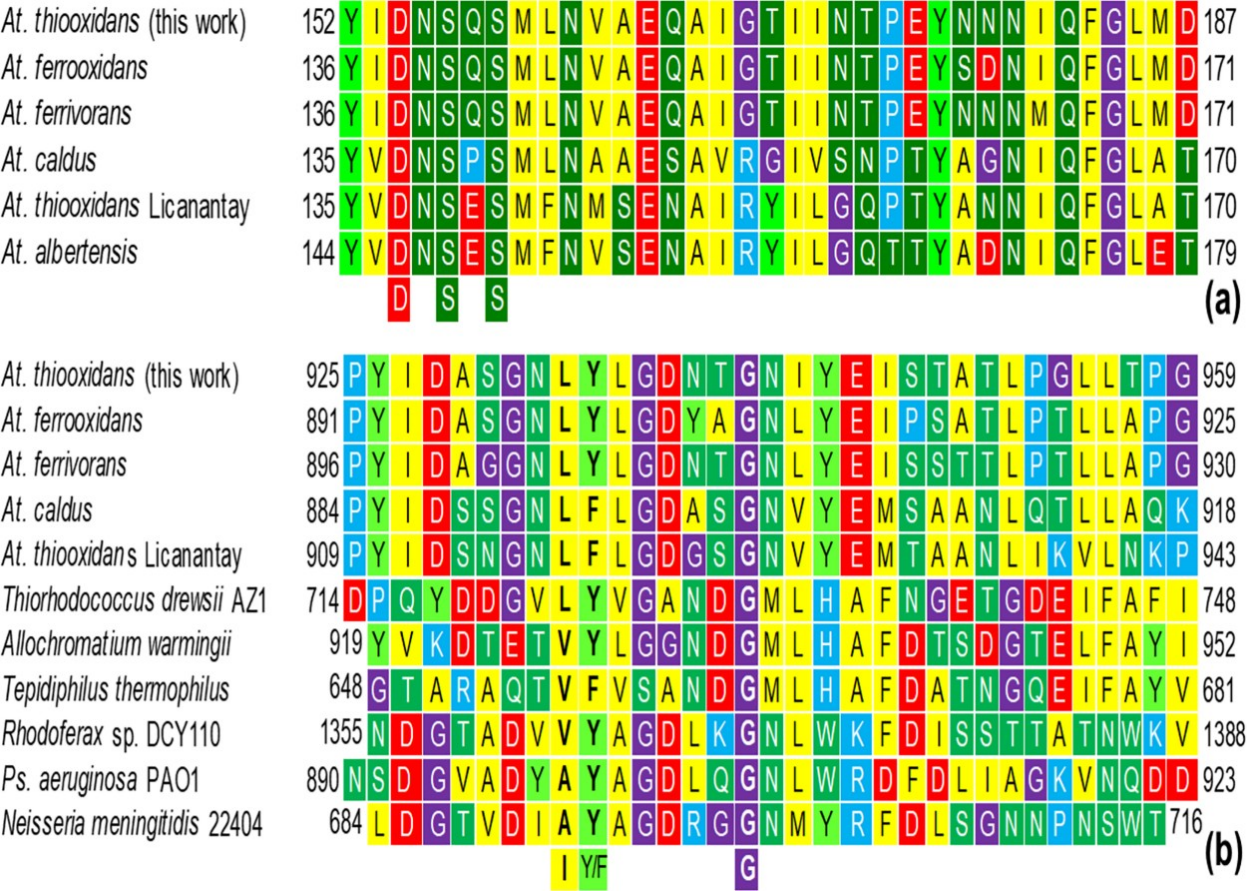
Multiple sequence alignment of PilY1 (AWP39905.1) of *At*. *thiooxidans*. (**a**) Partial view of the vWA domain within the NTD of some *Acidithiobacillus* spp. showing the MIDAS (DxSxS) motif. (**b**) The PQQ domain with the functional motif LYxxxxxG within the CTD of some *Acidithiobacillus* spp. and other bacteria with PilY1/PilC.

To gain insight into the functions of PilY, we performed several bioinformatic analyses using the aminoacidic sequence of PilY1 (AWP39905.1) we report here. First of all, such analyses confirm that PilY is a non-pilin protein, essentially a hydrophilic protein (gravy -0.081) whose last 462 aa (position 715 to1176) shares both sequence and structural homology with the C-terminal domain (CTD) of PilY1 of *Ps*. *aeruginosa* (3hx6.1) [24]; thus, PilY1 belongs to the conserved domain *Neisseria*-PilC superfamily [36]. However, the NTD of PilC from *Neisseria* spp, PilY1 from *Ps*. *aeruginosa* and PilY1 from *At*. *thiooxidans* (this report) are highly divergent sequences as they all show a rather poor homology. Nevertheless, both NTD and CTD of PilY1 from *At*. *thiooxidans* are highly similar with the TfP biogenesis protein from *Acidithiobacillus* spp. (Fig 3).

**Fig 4:**
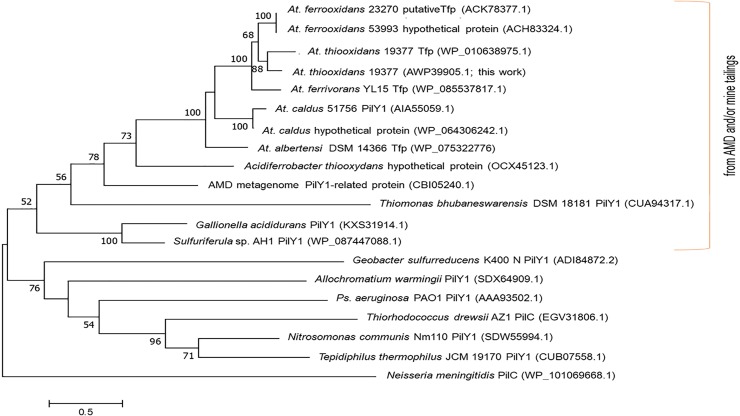
Molecular phylogenetic analysis of PilY1 of *At*. *thiooxidans* (AWP39905.1). The evolutionary history obtained by ML (log likelihood: (-16498.94) of PilY1/PilC, using 19 aa sequences and the PilY1 (AWP39905.1). There were 439 positions in the final dataset. Node numbers are support based on 1000 bootstrap replicates. Unrooted tree: no assumption was made regarding the ancestors of PilY1. Evolutionary analyses were conducted in MEGA7 [44]. Notes: AMD: acid mine drainage; Tfp: type IV pilin.

This can be better evidenced in the phylogenetic tree shown in Fig 4, which shows that using these sequences, the *Acidithiobacillus* genus forms a cohesive group, deeply related to iron- and/or sulfur-oxidizing microorganisms from acid mine drainage (AMD) or mine tailings such as *Th*. *bhubaneswarensis*, *Ac*. *thiooxydans*, *Ga*. *acididurans*, *Sulfuriferula* sp., and those of the AMD metagenome [47–51]. This phylogenetic tree also reveals that PilY1 and PilC are indeed homologues as all the analyzed sequences comprise the *Neisseria*-PilC beta-propeller domain. Thus, the phylogenetic analysis of PilY1/PilC suggests that nomenclature of these TfP protein, perhaps to the *Neisseria* spp. pilus assembly/adherence protein PilC.

**Fig 5:**
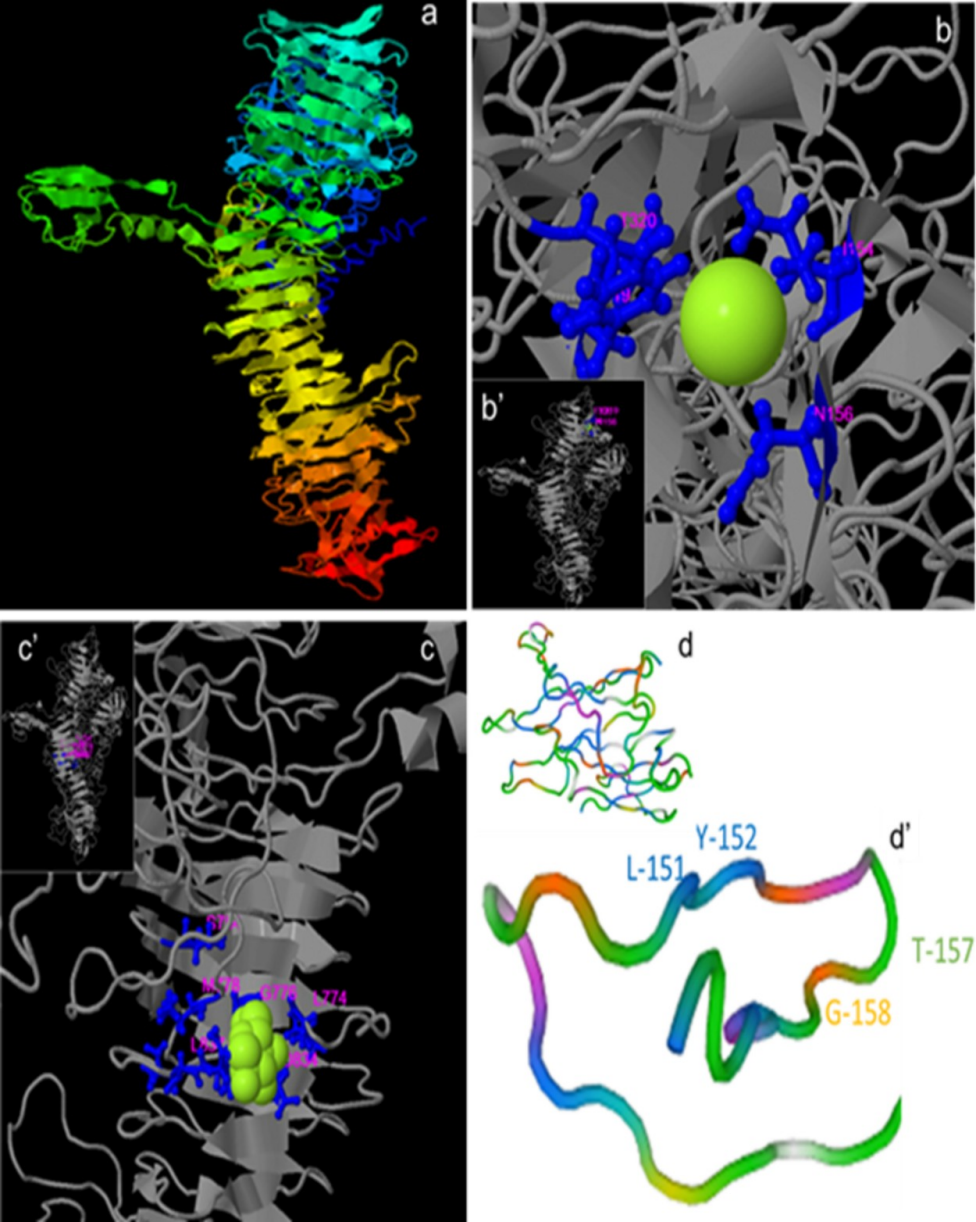
3-D models of the non-pilin protein PilY1 (AWP39905.1). The model shown has a TM-score of 0.61±0.14 with *Ps*. *aeruginosa* (TM-score >0.5 indicates a correct topology model, while TM-score <0.17 indicates a random similarity) **(a)**. A region of vWA showing the binding ligands L156, Y156, Y-319 and T-320 of MIDAS for Ca^2+^ binding (**b** and **b’**). a region CTD of PilY1/PilC (TM-score of 0.62±0.14) showing the binding residues for carbohydrates (*e*.*g*., *α*-D-glucose, β-glucose, α-dextrose) by S-754, L-774, G-776, M-778, D-834, L-835, Q-836, L-837 **(c** and **c’)**; 3-D modeling conducted in I-TASSER [38, 39] and BioLip [40]. Model of the PQQ region predicted by homology [37] and the anchor motif LYxxxxTG (**d** and **d’**).

The notion that PilY1 from these species belong to the very same conserved family as they share a high degree of homology, is further strengthened by a high degree of structural homology between them. The predicted 3-D model of PilY1 from *At*. *thiooxidans* (AWP39905.1), deduced based on structures reported to the PDB database (5J44A, 3HX6, 6EMKA, 3JA4A and 3IYlW), highlights the PilY1 CTD structure of *Ps*. *aeruginosa* (Fig 5) [24], from which shares a structural similarity of 22.73%, mainly among the CTD, which again, corresponds to the *Neisseria*-PilC beta-propeller domain of the tip-associated adhesin PilY1.

Further bioinformatic analysis of *At*. *thiooxidans* PilY1 (AWP39905.1) reveals the presence of five motifs also found in TfP proteins from *At*. *ferrivorans* SS3 (WP_085537817.1), *At*. *ferrooxidans* ATCC 53993 (ACH83324.1), *At*. *albertis* (WP_075322776), *At*. *caldus* MTH-04 (WP_064306242.1), and *At*. *thiooxidans* Licanantay (WP_031573362) (Figs 3 and 5). These motifs are:

- a von Willebrand factor type A (vWA) domain of 540 aa. This was found in the region from the aa 42 to the 581 and acquires an α/β Rossmann fold (alternating β-strand with α-helix). This vWA domain is involved in cellular functions such as cell migration, adhesion and signaling [36, 52].
- Within the vWA domain, a metal ion-dependent adhesion site (MIDAS motif, for Mn^2+^, Mg^2+^ or Ca^2+^) of 35 aa residues (region 153–187). This motif is folded as a short coil followed by an α-helix. MIDAS are commonly present in cell surface-adhesion receptors or molecules (CAMs), *e*.*g*., integrins [53]; thus, such components are involved in cell-cell and cell-matrix interactions through its adhesion function. MIDAS comprised the conserved components DxSxS, T, and D (Fig 3A) [36, 54]. After a modeling by homology, we found that the MIDAS motif of *At*. *thiooxidans* PilY1 (AWP39905.1) exhibits a 34.38% of similarity (covering 91%, from 2 to 33 aa) with the adhesive tip pilin of *Streptococcus agalactiae* (PDB: 3TW0) [52]. The constructed 3-D model specifies that the MIDAS motif within the vWA domain includes two different α-helix loops exposed on the protein surface (Fig 5B and 5B’), a location that allows its interaction with divalent metal ions such as Ca^2+^ via the binding ligands I-154, N-156 and T-320 of MIDAS.
- The aforementioned conserved CTD or *Neisseria*-PilC domain of 462 aa covering from position 715 to 1176. It is mainly composed of aliphatic aa (G, A, V, L and S, T; 60.2%) and slightly hydrophilic (gravy: -0.094). The 3-D model of this CTD (Fig 5C) was based only on structures of the CTD of *Ps*. *aeruginosa* PilY1 (PDB 3HX6) [24], showing a normalized Z-score of the threading alignments, up to 7.52. Other structural analogs predicted using I-TASSER, are oxidoreductases -substrate oxidation and electron transfer (PDB: 1H4JE, 1LRWA, 2D0VI, 1YIQA1, 1FLG), with Ca^2+^ or pyrroloquinoline quinone (PQQ) as ligands (see below).
- Within the *Neisseria*-PilC domain, the 3-D model also predicts binding sites for carbohydrates such as glucose (Fig 5C), which have been previously described for tail-spike proteins involved in recognition and adhesion of *Salmonella* and the *E*. *coli* bacteriophage HK620 [55].
- Also within the *Neisseria*-PilC domain, there is a motif that occurs in propellers of PQQ cofactor binding domains (SMART and InterPro accession numbers SM00564 and IPR018391, respectively). This motif covers 27 aa (from 926 to 952) and has been shown to catalyze redox reactions. This β-propeller repeat is found in enzymes with PQQ as cofactor in prokaryotic quinoprotein dehydrogenases that are involved in electron transfer processes, and thus energy transduction [56]. *Ps*. *aeruginosa* PilY1 (AAA93502) and *N*. *meningitidis* 22404 PilC (WP_101069668) also include the PQQ cofactor binding domain, while most of the *Acidithiobacillus* spp. and other sulfur-oxidizing microorganisms comprise the anchor motif LYxxxxTG (Fig 5B–5D). Interestingly, the iron- and/or sulfur-oxidizing microorganisms from AMD (*e*.*g*., *Th*. *bhubaneswarensis*, *Ac*. *thiooxydans*, *Ga*. *acididurans*, *Sulfuriferula* sp.) also comprise the PQQ domain (Fig 3B), while the PilY1-related protein (fragment) from the AMD metagenome (CBI05240.1) mostly corresponds to the vWA domain within the NTD that also comprises the MIDAS motif, DxSxSxxxxxxxxxxT (Fig 3A). Furthermore, according to SMART analyses, the other sulfur-oxidizing microorganisms presented in the phylogenetic tree shown in Fig 4 (*Te*. *thermophilus* and *Th*. *drewsii* AZ1) also include the PQQ domain in their tip-associated adhesin PilY1/PilC [33]. Thus, the presence of the PQQ motif in such PilY1/PilC proteins suggests that this adhesin initiates the biooxidation of reduced compounds (*e*.*g*., S^0^ or metal sulfides as FeS_2_, and CuFeS_2_) and may be involved in electron transfer between the substrate and other components. *Sensu* Li and Li [28] suggest that biooxidation of Fe(III) by *At*. *ferrooxidans* depends on functional pili, which transfer electrons from the reduced Fe to the cell surface, while attaches strongly to solid surfaces such as FeS_2_ [6].

#### Pilins PilW and PilV

**Fig 6:**
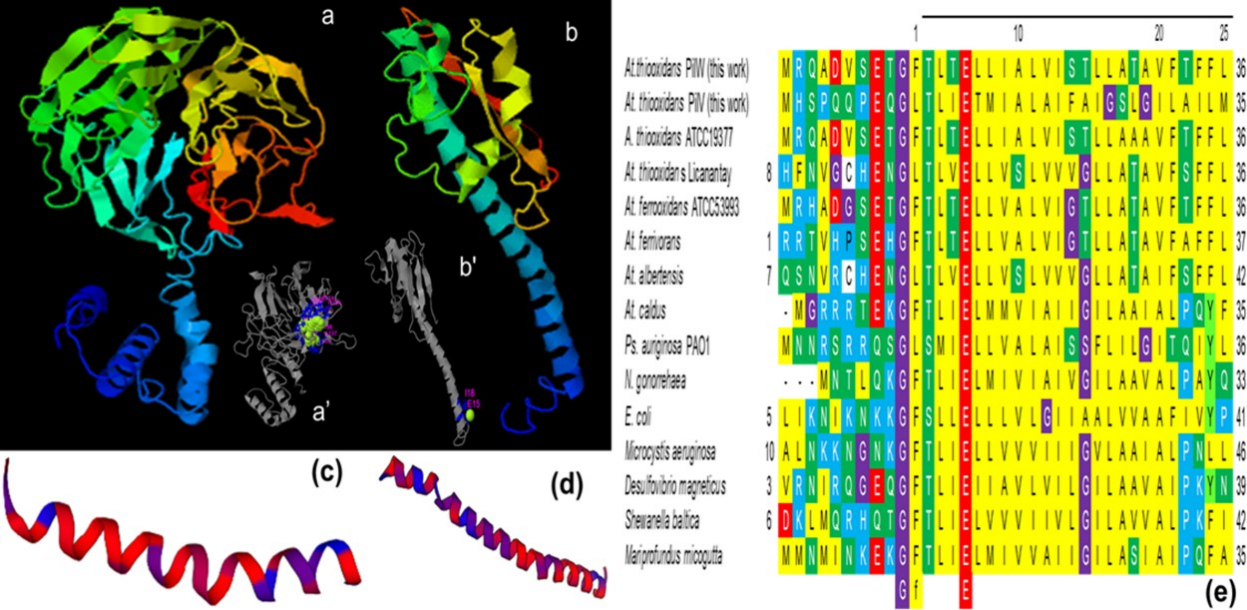
Bioinformatic analyses of the confirmed sequences of *At*. *thiooxidans* PilW (AWP39906.1) and PilV (AWP39907.1). (**a**) 3-D model of PilW (TM-score: 0.42±0.14) and (**b**) PilV (TM-score: 0.49±0.15) conducted in I-TASSER [38, 39] and BioLip [40]; the models were obtained based on structures from the PDB data base; for PilW: 5K8G, 1OQW, 4UT1A, 3SOK, 4M00, 5GAOE; for PilV: 1OQW, 3SOK, 5BW0, 1AY2 AND 3CI0. Modeling by homology of the NTD of (**c**) PilW (1–81 aa) and (**d**) PilV (11–62 aa) using as template the 3nje.1 minor pseudopilin from the *Ps*. *aeruginosa* [15]. for PilW, and the 5vxy.1.E pilin of *Ps*. *aeruginosa* and *N*. *gonorrhoeae* [57, 58] for PilV. (**e**) Multiple sequence alignment conducted in MEGA7 [44], of the region NTD (25–28 aa) of the confirmed PilW (AWP39906.1) and PilV (AWP39907.1) sequences of *At*. *thiooxidans*, and of other sequences from the gene bank of prepilin containing proteins of *At*. *thiooxidans* ATCC 19377 (WP_010638979.1), *At*. *thiooxidans* Licanantay (WP_051690664.1). *At*. *ferrooxidans* ATCC 593993 (ACH83326.1), *At*. *ferrivorans* (WP_035191506.1), *At*. *albertensis* (WP_075323115.1) and *At*. *caldus* (WP_070113768.1), as well as TfP pilins or prepilins of *Ps*. *auriginosa* PAO1 (NP_253215.1) *N*. *gonorrehaea* (SBO76855.1), *E*. *coli* (PIM50236.1), *Microcystis aeruginosa* (WP_012267210.1), *Desulfovibrio magneticus* (EKO40131.1), *Shewanella baltica* (WP_012588380.1) and *Mariprofundus micogutta* (WP_083530569.1).

The 3D-modeling of PilW and PilV of *At*. *thiooxidans* (Fig 6A and 6B) reveals high similarities of these two proteins to the pilin of *Ps*. *aeruginosa* strain K, PAK (PDB 1OQW). For PilW, the model was also based on structures of a protein with structural similarity to the flagellin protein of *Burkholderia pseudomallei* (PDB 4UT1A), a putative peptide-binding domain (adhesin) of *Marinomonas primoryensis* (5K8G), and the pilin FimA for adhesion from *Dichelobacter nodosus* (3SOK).

Likewise, the 1OQW and 3SOK sequences, PilW and PilV from *At*. *thiooxidans* and other TfP pilins have leader peptides of approximately 20–25 aa in the NTD with a highly conserved G at the start of the GFXXXXE domain (Fig 6E). According to Dalrymple and Mattick [59] and Mattick [60], all the necessary information for the processing of subunits and assembly of pili is located within these initial residues of the α1-N; thus, mutations of NTD residues can markedly affect pilus assembly [13]. Specifically, the conserved glutamic acid E5 is the only charged residue in NTD (Fig 6B’), which is essential for the pilus assembly and seems to be required for the methylation step [36, 57, 61]. In *At*. *thiooxidans*, E5 interacts with divalent cations such as Ca^2+^ (Fig 6B).

The CTD of PilW includes a region from the aa 239 to 371 which is identified as the “TfP pilus assembly protein PilW” (pfam16074). Its predicted secondary structure is mainly strands and coils, that define a globular head (Fig 6A). In addition, a globular head is also observed in the 3-D model of PilV (Fig 6B). This globular head domain is a structurally variable region among the different pili, this variability imposes different functions, *e*.*g*., specific subunit interactions and packing arrangements within the filament, pilus-pilus interaction, and interactions between the pili and their environment [57].

**Fig 7:**
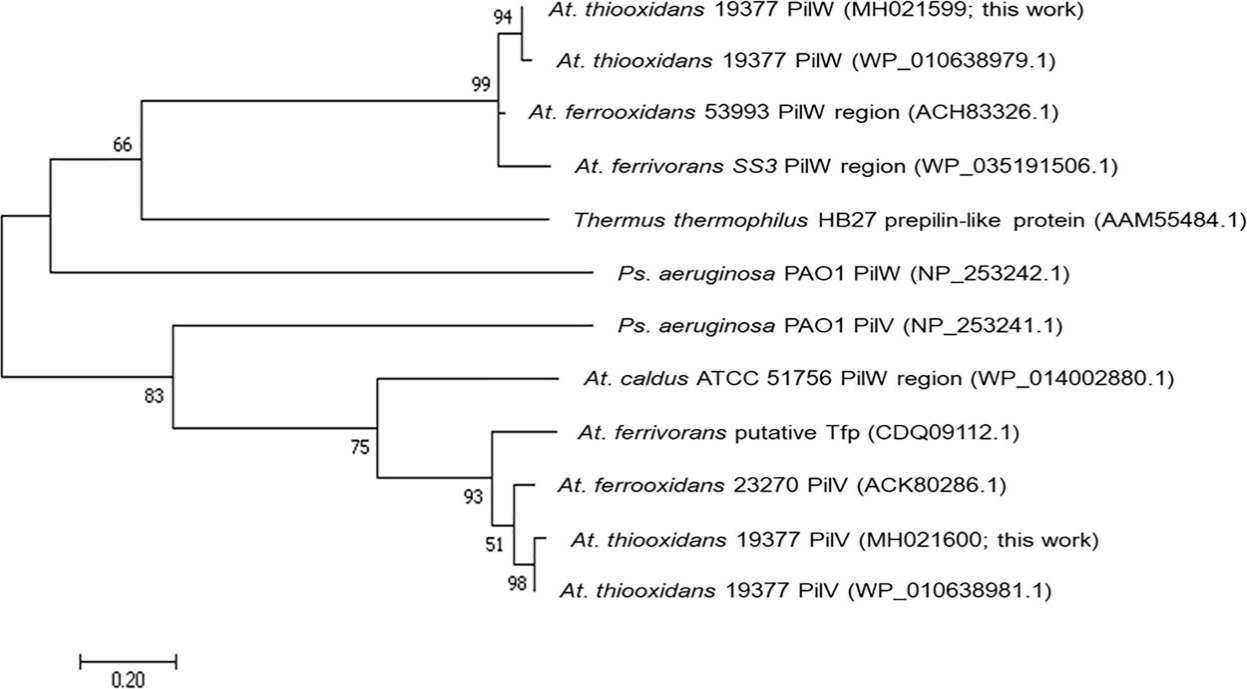
Phylogenetic relationships based on the pilins PilW (AWP39906.1) and PilV (AWP39907.1) of *At*. *thiooxidans*. The evolutionary history obtained by ML (log likelihood -1678.78) using 12 amino acids sequences. There was a total of 97 positions in the final dataset. Node numbers are support based on 1000 bootstrap replicates. Unrooted tree: no assumption was made regarding the ancestors of PilW and PilV. Evolutionary analyses were conducted in MEGA7 [44].

The molecular phylogenetic analysis confirms that PilW and PilV from *At*. *thiooxidans* are core subunits of TfP, similar to PilW and PilV of *Ps*. *aeruginosa* PAO1 (Fig 7).

Summarizing, PilY1 (AWP39905.1) comprises the conserved *Neisseria*-PilC (superfamily) beta-propeller domain of the tip-associated adhesin, whereas PilW (AWP39906.1) and PilV (AWP39907.1) are part of the superfamily of putative TfP assembly proteins. Further analysis is required to elucidate the specific function of PilY1, PilW and PilV as well as the molecular mechanisms of pili assembly in *At*. *thiooxidans*. Current undergoing work in our laboratory intends to evaluate through gene silencing the role of PilY1 in the ability to attach to mineral surfaces.

## Supporting information

S1 Fig (DOCX)

S1 Table (DOCX)

S2 Table (DOCX)
