# Supplementary material for: Sequence analysis and confirmation of the type IV pili-associated proteins PilY1, PilW and PilV in Acidithiobacillus thiooxidans
Source: PLoS One. 2019 Jan 7;14(1):e0199854. doi: 10.1371/journal.pone.0199854 (PMC6322766; doi:10.1371/journal.pone.0199854)

**S1 Figure.**

Pili coding sequences of *At. thiooxidans*. The coding regions for each pilus was amplified by RT-PCR using several pair of primers. Each PCR product was purified and sequenced. The whole sequence was assembled using overlapping regions and compared against their respective annotated genomic sequence reported to GenBank: PilY1 mRNA sequence (GenBank MH021598.1), PilW mRNA sequence (MH021599.1) and, PilV mRNA sequence (MH021600.1). Changes versus the annotated sequence are noted as follows: letters on top of the sequence indicate individual base changes whereas insertions are pointed out in red within the sequence and deletions are shown as blue hyphens. Black hyphens on top of the red insertions represent the absence of that sequence in the annotated sequence while the letters on top of the blue hyphens represents the deleted regions within our sequence.


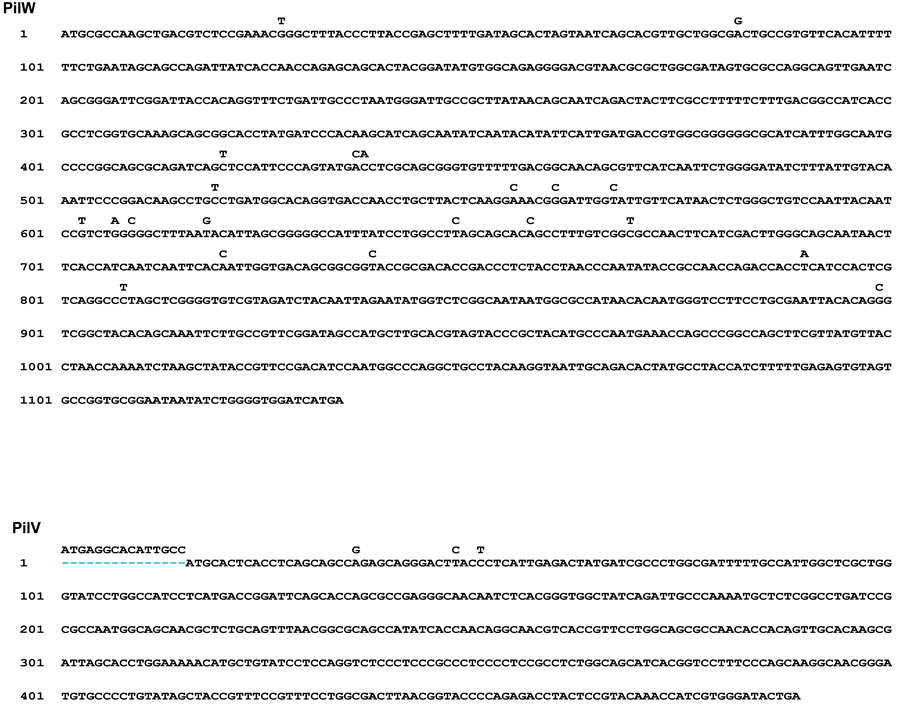


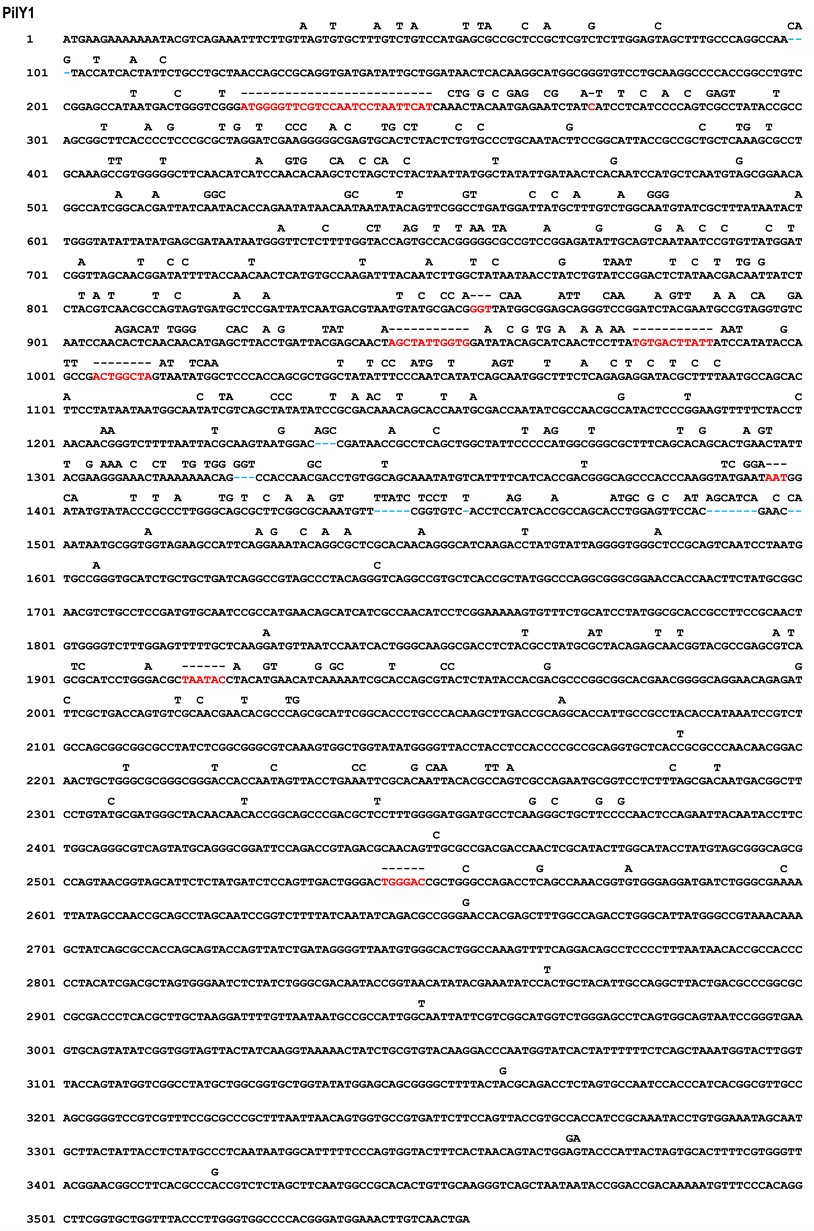

Supplement: S1 Fig — (DOCX) [file pone.0199854.s001.docx]
