# Supplementary material for: Sequence analysis and confirmation of the type IV pili-associated proteins PilY1, PilW and PilV in Acidithiobacillus thiooxidans
Source: PLoS One. 2019 Jan 7;14(1):e0199854. doi: 10.1371/journal.pone.0199854 (PMC6322766; doi:10.1371/journal.pone.0199854)
Supplement: S1 Table — (DOCX) [file pone.0199854.s002.docx]

## S1 Table

Selected proteins of the TfP of *At. thiooxidans* ATCC 19377 (NZ_AFOH00000000.1; Valdés *et al*. 2011).

| **Pilin** | **Locus** | **Access number** | **Characteristics** |
| --- | --- | --- | --- |
| type IV pilin biogenesis protein (PilY1) | ATHIO_RS0106065 | WP_010638975.1 | Non-pilin protein. Region 401-559 aa: the von Willebrand factor type A (vWA) as a conserved protein domain family^1^ |
| Prepilin-type N-terminal cleavage/methylation domain-containing protein (PilW) | ATHIO_RS0106075 | WP_010638979.1 | Adhesion, outer-membrane protein. Region 10-33 aa: Type IV pilin N-term methylation site GFxxxE (pfam13544)^1^. Region 239-371 aa: TfP pilus assembly protein PilW (pfam16074)^1^ |
| Prepilin-type N-terminal cleavage/methylation domain-containing protein (PilV)* | ATHIO_RS0106080 | WP_010638981.1 | Region 6-162 aa: TfP pilus assembly protein PilV. Cell motility, extracellular structures |
