# Supplementary material for: Sequence analysis and confirmation of the type IV pili-associated proteins PilY1, PilW and PilV in Acidithiobacillus thiooxidans
Source: PLoS One. 2019 Jan 7;14(1):e0199854. doi: 10.1371/journal.pone.0199854 (PMC6322766; doi:10.1371/journal.pone.0199854)
Supplement: S2 Table — (DOCX) [file pone.0199854.s003.docx]

**S2 Table**

Primers for *pilY1*, *pilW* and *pilV* of *At. thiooxidans* ATCC 1937. As a positive control of each PCR reaction, we designed a pair of primers that amplify 598 bp of *16S* rRNA (AJ459803.1).

| **Primer** | **Sequence (5' → 3')** | **Length (nt)** | **Amplicon length (bp)** |
| --- | --- | --- | --- |
| *pilY-1 up* | CCATTTTGAGGAAAGTGCAAT | 21 | 190 |
| *pilY-1 lw* | CGCCATGCCTTGTGAGTT | 18 |  |
| *pilY-2 up* | AGGTGATGATATTGCTGGATAACTC | 25 | 208 |
| *pilY-2 lw* | GTATTGCAGGGGACGGAGTA | 20 |  |
| *pilY-3 up* | TACTCCGTCCCCTGCAATAC | 20 | 794 |
| *pilY-3 lw* | GCGTTGGCGATATTGGTC | 18 |  |
| *pilY-4 up* | CATTTTCATCACCGATGGG | 19 | 384 |
| *pilY-4 lw* | ATGATGCTGTTCATGGCG | 18 |  |
| *pilY-5 up* | CAACTTCTATGCGGCAACG | 19 | 459 |
| *pilY-5 lw* | CCCATATACCAGCCACTTTGA | 21 |  |
| *pilY 6 up* | CATTGCCGCCTACACCAT | 18 | 541 |
| *pilY-6 lw* | AGGCTGCGGTTGGCTATA | 18 |  |
| *pilY-7 up* | CGCCAGTAACGGTAGCATTC | 20 | 507 |
| *pilY-7 lw* | ATATACTGCACTTCACCCGGA | 21 |  |
| *pilY-8 up* | AATTATTCGTCGGCATGGTC | 20 | 467 |
| *pilY-8 lw* | GTGAAGGCCGTTCCGTAA | 18 |  |
| *pilY-9 up* | GGGGTTCGTCCAATCCTAA | 19 | 795 |
| *pilY-9 lw* | CCATATTACTAGCCAGTCGGCT | 22 |  |
| *pilY-10 up* | CGTAATGTATGCGACGGGT | 19 | 643 |
| *pilY-10 lw* | ATTGTTCGTGGAACTCCAGG | 20 |  |
| *pilY-11 up* | AATGGATATGTATACCCGCCC | 21 | 514 |
| *pilY-11 lw* | AGGTATTAGCGTCCCAGGATG | 21 |  |
| *pilWsp* up* | TTTACCCTTACCGAGCTTTTGAT | 23 | 1104 |
| *pilWsp* lw* | TCATGATCCACCCCAGATATTA | 22 |  |
| *pilW-1 up* | ATGCGCCAAGCTGACGTC | 18 | 1134 |
| *pilW-1 lw* | TCATGATCCACCCCAGATATTA | 22 |  |
| *pilW-2 up* | CCCCAGAGACCTACTCCGTA | 20 | 186 |
| *pilW-2 lw* | TAGTGCTGCTCTGGTTGGTG | 20 |  |
| *pilW-3 up* | CCAGGCTGCCTACAAGGTAA | 20 | 116 |
| *pilW-3 lw* | TAGGAAACGCTTTGCTTGCT | 20 |  |
| *PilW-4 up* | AGACTACTTCGCCTTTTTCTTTGA | 24 | 869 |
| *pilW-4 lw* | TCATGATCCACCCCAGATATTA | *22* |  |
| *pilVsp* up* | CTCACTCTCATTGAGACTATGATCG | 25 | 444 |
| *pilVsp* lw* | TCAGTATCCCACGATGGTTTG | 21 |  |
| *pilV-1 up* | ATGCACTCACCTCAGCAGC | 19 | 47 |
| *pilV-1 lw* | TCAGTATCCCACGATGGTTTG | 21 |  |
| *pilV-2 up* | GCAACCTAAAATGAGGCACATT | 22 | 269 |
| *pilV-2 lw* | CGTTGCCTGTTGGTGATATG | 20 |  |
| *pilV-3 up* | CATTGGCTCGCTGGGTAT | 18 | 432 |
| *pilV-3 lw* | AGTTTCGGAGACGTCAGCTT | 20 |  |
| *16s up* | CGCTGGAAGAGGAGCCTAC | 21 | 598 |
| *16s lw* | TTTAGGGCGTGGACTACCAG | 20 |  |

* sp: without propeptide
